# Three-point traction functions like full-face traction: facilitating rapid excision of a laterally spreading tumor

**DOI:** 10.1055/a-2512-0861

**Published:** 2025-01-21

**Authors:** Hui Liu, Zhi Guo Wang

**Affiliations:** 1 Department of Gastroenterology, Second Affiliated Hospital of Dalian Medical University, Dalian, China; 2 Department of Gastroenterology, Air Force Medical Center, Air Force Medical University, Beijing, China

With the progression of technology, endoscopic submucosal dissection (ESD) has been increasingly applied in clinical practice. However, ESD still faces technical challenges in certain cases. To reduce the difficulty of the procedure, shorten the operation time, and decrease the incidence of complications, various traction devices have been invented and employed clinically 1 2 3 . Additionally, owing to limitations of single-point traction, multipoint traction techniques have been developed to achieve more efficient dissection. These multipoint traction techniques represent technological improvements and innovations based on traction devices 4 5 . Herein, we introduce a novel multipoint traction method that enhances traction force, enabling rapid dissection of the lesion.

A 55-year-old woman underwent colonoscopy, during which a laterally spreading tumor measuring approximately 2.2 × 1.8 cm was identified in the mid-transverse colon ( Fig. 1 **a** ). Subsequently, she underwent ESD ( Video 1 ). ESD was considered challenging due to the lesion extending over intestinal folds and being influenced by arterial pulsation. Therefore, we employed a novel multipoint traction method to address these difficulties.

**Fig. 1:**
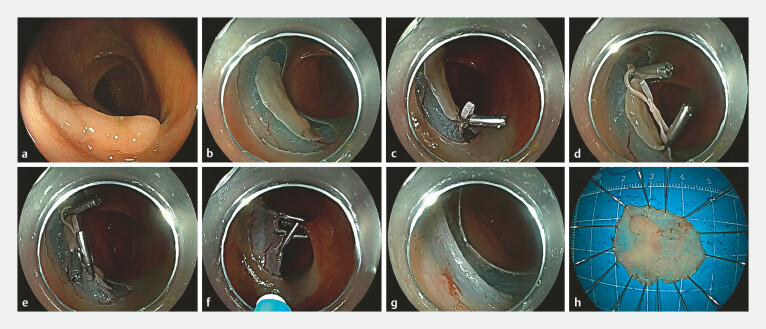
Multipoint traction for the treatment of colonic laterally spreading tumor (LST). **a** An LST measuring approximately 2.2 × 1.8 cm was identified in the mid-transverse colon. **b** Following submucosal injection, a circumferential incision was made around the lesion. **c** One loop of an “8”-shaped rubber ring was secured to the right side of the lesion using a clip. **d** The opposite loop of the “8”-shaped rubber ring was anchored to the left side of the lesion with another clip. **e** A third clip was employed to fix the central part of the rubber ring at the anal side of the lesion. **f** A fourth clip was applied to secure the middle part of the rubber ring to the opposing intestinal wall. **g** Complete dissection of the submucosal layer of the lesion was achieved. **h** Post-ESD appearance of the resected lesion.

Multipoint traction for the treatment of colonic laterally spreading tumor.Video 1

First, a circumferential mucosal incision was made using a mucosal cutting knife ( Fig. 1 **b** ). An elastic rubber ring was then knotted in the middle to form an “8” shape. One loop of the rubber ring was secured to the right side of the lesion using a clip ( Fig. 1 **c** ). A second clip was used to anchor the opposite loop of the rubber ring to the left side of the lesion ( Fig. 1 **d** ). The central part of the rubber ring was then fixed at the anal side of the lesion with a third clip ( Fig. 1 **e** ). A fourth clip was applied to the middle of the rubber ring, securing it to the opposite intestinal wall ( Fig. 1 **f** ). Following this traction technique, the submucosal layer was clearly exposed, transitioning from point traction to surface traction. This increased the traction area and achieved a converging effect. Benefiting from the tension generated by the traction device, the endoscopist successfully completed the dissection of the entire submucosal layer within 2 minutes ( Fig. 1 **g** ), without causing damage to the muscularis propria. The resected lesion measured approximately 2.8 × 2.2 cm ( Fig. 1 **h** ). Postoperative pathology revealed a tubular adenoma with low grade dysplasia, with no tumor tissue observed at the horizontal and basal margins.

This novel multipoint traction method not only converges the lesions from both sides but also utilizes three traction points to pull the lesion toward the opposite side of the intestinal lumen, creating a fan-like convergence. This approach offers two primary advantages: it reduces the area of the lesion that needs to be dissected and ensures consistent tension throughout the submucosal layer, facilitating rapid dissection. However, we are considering whether an alternative setup with the third clip securing both loops at the anal side of the lesion, or the fourth clip similarly securing both loops on the opposite intestinal wall, might yield even better results. We will continue to explore and study this possibility.

In summary, our new multipoint traction method provides valuable insights for endoscopists.

Endoscopy_UCTN_Code_TTT_1AQ_2AD_3AD

## Notes

Endoscopy E-Videos https://eref.thieme.de/e-videos
*E-Videos* is an open access online section of the journal *Endoscopy* , reporting on interesting cases and new techniques in gastroenterological endoscopy. All papers include a high-quality video and are published with a Creative Commons CC-BY license. Endoscopy E-Videos qualify for HINARI discounts and waivers and eligibility is automatically checked during the submission process. We grant 100% waivers to articles whose corresponding authors are based in Group A countries and 50% waivers to those who are based in Group B countries as classified by Research4Life (see: https://www.research4life.org/access/eligibility/ ). This section has its own submission website at https://mc.manuscriptcentral.com/e-videos .
